# Specific Syndecan-1 Domains Regulate Mesenchymal Tumor Cell Adhesion, Motility and Migration

**DOI:** 10.1371/journal.pone.0014816

**Published:** 2011-06-23

**Authors:** Fang Zong, Eleni Fthenou, Filip Mundt, Tünde Szatmári, Ilona Kovalszky, László Szilák, David Brodin, George Tzanakakis, Anders Hjerpe, Katalin Dobra

**Affiliations:** 1 Division of Pathology, Department of Laboratory Medicine, Karolinska Institutet, Stockholm, Sweden; 2 Division of Morphology, Department of Histology, School of Medicine, University of Crete, Heraklion, Greece; 3 1st Institute of Pathology and Experimental Cancer Research, Semmelweis University, Budapest, Hungary; 4 Institute of Biology, Savaria University Center, Western Hungarian University, Szombathely, Hungary; 5 Bioinformatics and Expression Analysis Core Facility, Department of Biosciences and Nutrition, Karolinska Institutet, Stockholm, Sweden; University of Colorado, Boulder, United States of America

## Abstract

**Background:**

Syndecans are proteoglycans whose core proteins have a short cytoplasmic domain, a transmembrane domain and a large N-terminal extracellular domain possessing glycosaminoglycan chains. Syndecans are involved in many important cellular processes. Our recent publications have demonstrated that syndecan-1 translocates into the nucleus and hampers tumor cell proliferation. In the present study, we aimed to investigate the role of syndecan-1 in tumor cell adhesion and migration, with special focus on the importance of its distinct protein domains, to better understand the structure-function relationship of syndecan-1 in tumor progression.

**Methodology/Principal Findings:**

We utilized two mesenchymal tumor cell lines which were transfected to stably overexpress full-length syndecan-1 or truncated variants: the 78 which lacks the extracellular domain except the DRKE sequence proposed to be essential for oligomerization, the 77 which lacks the whole extracellular domain, and the RMKKK which serves as a nuclear localization signal. The deletion of the RMKKK motif from full-length syndecan-1 abolished the nuclear translocation of this proteoglycan. Various bioassays for cell adhesion, chemotaxis, random movement and wound healing were studied. Furthermore, we performed gene microarray to analyze the global gene expression pattern influenced by syndecan-1. Both full-length and truncated syndecan-1 constructs decrease tumor cell migration and motility, and affect cell adhesion. Distinct protein domains have differential effects, the extracellular domain is more important for promoting cell adhesion, while the transmembrane and cytoplasmic domains are sufficient for inhibition of cell migration. Cell behavior seems to depend also on the nuclear translocation of syndecan-1. Many genes are differentially regulated by syndecan-1 and a number of genes are actually involved in cell adhesion and migration.

**Conclusions/Significance:**

Our results demonstrate that syndecan-1 regulates mesenchymal tumor cell adhesion and migration, and different domains have differential effects. Our study provides new insights into better understanding of the role of syndecans in tumor progression.

## Introduction

Tumor cell invasion of surrounding tissue is one key factor for tumor aggressiveness and is dependent on the complex interplay of tumor cell adhesion, motility and migration. Tumor cells must first adhere to extracellular matrix (ECM) and cell surface molecules as they invade. Cell motility and migration are dynamic processes that require continuous assembly and disassembly of cell-cell and cell-matrix adhesions [1], since these cell behaviors are consequences of the interactions between tumor cells and their surrounding microenvironment. Among the many interacting cellular molecules, syndecans are emerging as important regulators for these processes and thus crucial for tumor invasion.

Syndecans are a family of transmembrane proteoglycans (PGs) consisting of a type I membrane core protein with glycosaminoglycan (GAG) chains covalently attached to the extracellular portion of protein core [2]. In mammals, there are four syndecan members transcribed from four genes. According to the similarities in core protein structure and GAG chain substitution they are divided into two sub-families: syndecan-1 and -3, and syndecan-2 and -4. All syndecans have a large extracellular domain (EC domain), a single transmembrane domain and a short cytoplasmic domain, each of which contributes to syndecan function [3], [4], [5].

The N-terminal EC domains are different in each syndecan with the exception of the conserved GAG attachment sites. The EC domains of syndecan-1, -2 and -4 have all been associated with cell adhesion [6], [7], for a review see reference [8]. Shedding of the EC domain occurs at protease sensitive sites close to the cell membrane. One identified cleavage site is G245-L246, about 7 amino acids from the cell membrane in human syndecan-1 [9]. Another juxtamembrane cleavage site is identified to amino acids A243 and S244 in murine syndecan-1, which sequence equivalent is present in human syndecan-1. It is speculated that cleavage at the A243-244 maybe partly utilized in human syndecan-1 [10], [11], [12].

The transmembrane domain is highly conserved among the four syndecan family members. The GXXXG motif positioned very close to the cell membrane promotes homo- and hetero-dimerizations of syndecans, thus characterizing the transmembrane domain as essential for the activation of the cytoplasmic domain and downstream signaling [13], [14], [15]. For syndecan-3 oligomerization both the transmembrane domain and the adjacent EKRE motif of the EC domain are needed [13]. The function of the corresponding DRKE sequence in syndecan-1 molecule is still not clear.

The cytoplasmic domain contains two highly conserved regions (C1 and C2), which are identical in all four syndecans (with the exception of a R for K substitution in C1 of syndecan-2). The cytoplasmic domains bind cytoskeletal and PDZ-domain proteins through the C1 and C2 regions, respectively, and thus regulate dynamics of the actin cytoskeleton and membrane trafficking. These interactions control syndecan recycling through endosomal compartments, promote internalization of accompanying protein cargo, and regulate cell adhesion and various signaling systems (For a review see references [14], [15], [16]). The central variable region (V), flanked by C1 and C2, is unique for each family member. The V region of syndecan-1 plays an essential role in lamellipodial spreading, actin bundling and cell migration [17].

Syndecan-1 is present not only on the cell surface but also at intracellular locations [18], [19], [20]. In particular, it accumulates in the nucleus in a time-and tubulin-dependent manner [18]. We were the first to show that the RMKKK motif present at the juxtamembrane region of the cytoplasmic domain, may serve as a nuclear localization signal (NLS) guiding syndecan-1 to the nucleus and, in parallel, decreases the proliferation of two mesenchymal tumor cell lines [21], [22].

Publications on myeloma [23], [24] and mammary carcinoma models [25], [26] point to a syndecan-1 structure-function relationship in tumor cell adhesion and migration. A stretch of 5 hydrophobic amino acids, AVAAV (amino acids 222–226) within the EC domain close to the plasma membrane, was identified to be critical forsyndecan-1-mediated inhibition of cell invasion [27]. Syndecan-1 can cooperate with integrins to regulate adhesion-complex formation, cytoskeletal organization and cell spreading and directional migration [28]. Recently, a site for direct binding and activating integrins, without the apparent involvement of GAG chains [7], has been mapped to a region of the EC domain (amino acids 88–121) in syndecan-1 (For a review see reference [8]). However, most studies of syndecans on cell adhesion and migration have been focused on syndecan-4 and syndecan-2 (For a review see references [4], [29], [30]). The role of syndecans in tumor development may vary with tumor stage and type. Information on syndecan-1 involvement in mesenchymal tumor cell adhesion and migration is still lacking, especially with regard to the specific contribution of its distinct functional domains.

In the light of our recent findings, showing that full-length syndecan-1 and its specific domains inhibit mesenchymal tumor cell proliferation [22], we investigate, in the present study, its role in cell adhesion and migration. We modulated syndecan-1 expression levels in two mesenchymal tumor cell lines: a human fibrosarcoma B6FS cell line and a human malignant mesothelioma (MM) STAV-AB cell line. We examined subsequent effects on different parameters associated with cell adhesion, motility and migration, focusing not only on the contribution of the specific syndecan-1 domains, but also on the expression levels of these constructs.

## Results

### Generation of strong and weak syndecan-1 expressers

**Figure 1:**
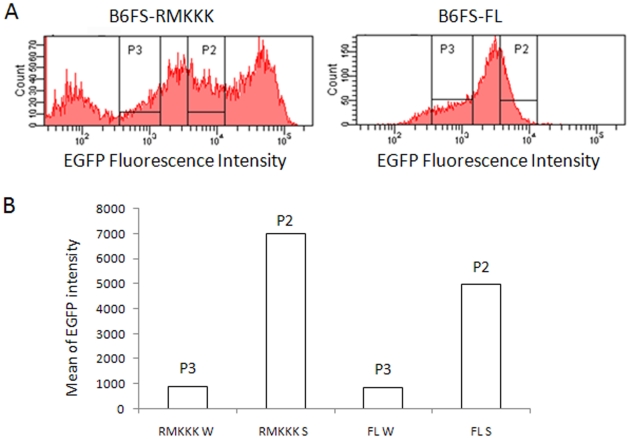
Strong and weak expressers generated by FACS sorting. B6FS stable transfectants of the full-length syndecan-1/EGFP and the RMKKK/EGFP were separated by using FACS sorting based on their EGFP intensity. The subpopulations with high (P2) or low (P3) EGFP intensity were isolated and subcultured as strong or weak expressers respectively. A. FACS plots showing the separation of the subpopulations. B. Mean value of EGFP intensity of the subpopulations. As for the suffix for RMKKK and FL, “W” stands for weak expresser while as “S” stands for strong expresser.

B6FS stable transfectants of the full-length syndecan-1/EGFP and the RMKKK/EGFP were separated into two subpopulations based on their EGFP fluorescence intensity by using FACS sorting. The subpopulations with high or low EGFP intensity were separated and isolated (Figure 1A), and sub-cultured into strong or weak expressers, respectively. The EGFP intensity of the RMKKK strong expresser (RMKKK S) was about 8 fold higher than the RMKKK weak expresser (RMKKK W); the full-length strong expresser (FL S) had EGFP intensity 6 fold higher than the full-length weak expresser (FL W) (Figure 1B). FACS sorting was also performed on the STAV-AB stable transfectants of the full-length syndecan-1/EGFP and the RMKKK/EGFP. The subpopulations with high or low EGFP intensity were obtained (data not shown), but they failed to grow out in culture.

### Effects of syndecan-1 on cell adhesion

**Figure 2:**
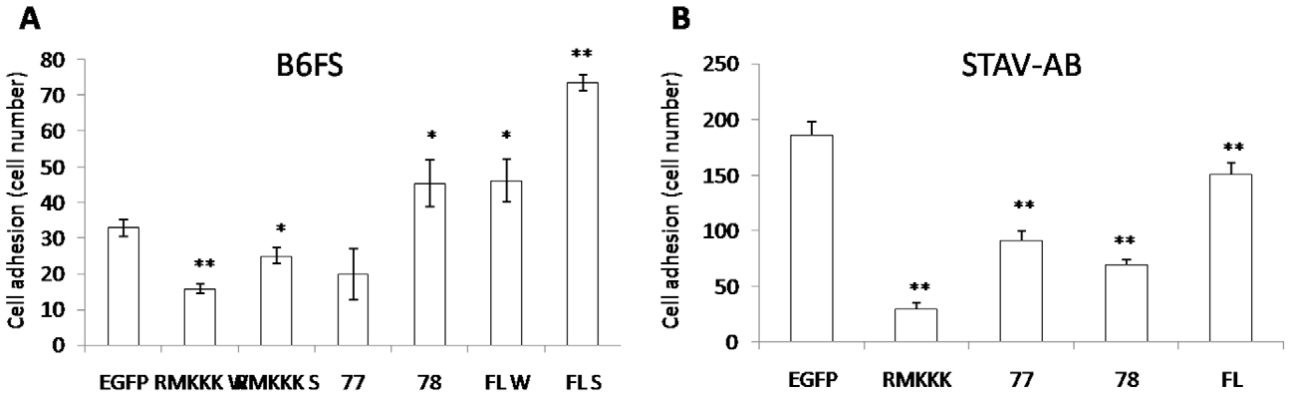
Effect of syndecan-1 overexpression on *mesenchymal* cell adhesion. Stable transfectants were serum starved for 24 h before seeding into a black 96 well plate in RPMI with 10% of the respective sera. After 5 min the floating cells were washed away with culture medium. The numbers of the attached cells were measured using a fluorometer. All analyses were performed in triplicate. The reported values are Means ± SD. Statistical significance: ^*^ *p*<0.01, ^**^ *p*<0.001, compared to EGFP control.

Overexpression of the full-length syndecan-1 enhanced fibrosarcoma cell adhesion, and the high expresser gave a more pronounced effect (Figure 1 and Figure 2A), suggesting that syndecan-1 stimulates cell adhesion in a dose-dependent manner. The 78 transfection marginally enhanced cell adhesion, while the 77 construct had no significant effect. The RMKKK construct, on the other hand, reduced cell adhesion. As for the effect on the STAV-AB cell line, cell adhesion was hampered to some extent by the full-length construct (Figure 2B). A common feature in both cell lines was that cell adhesion was dependent mainly on the EC domain and seemed to correlate to the size of the construct.

### Syndecan-1 inhibits cell chemotaxis

**Figure 3:**
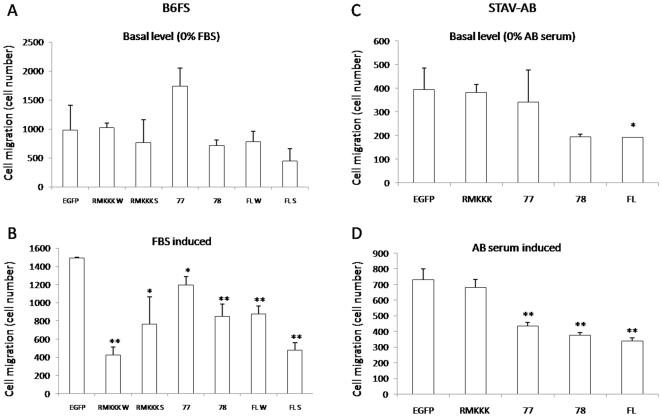
Effect of syndecan-1 overexpression on *mesenchymal* cell chemotaxis. Stable transfectants were serum starved for 24 h before seeding into the upper well of a two chamber system with serum-free medium. Medium containing 10% of the respective sera was placed in the bottom chamber. After 6 h incubation, those cells that had migrated to the bottom of the membrane insert were dislodged and their number measured using a fluorometer. All analyses were performed in triplicate. The reported values are Means ± SD. Statistical significance: ^*^ *p*<0.01, ^**^ *p*<0.001, compared to EGFP control.

Further, we studied whether overexpression of syndecan-1 may affect the basal and chemoattractant-induced mesenchymal tumor cell migration. While the basal level of cell migration was not affected by transfection of the full-length syndecan-1 construct in fibrosarcoma cells, FBS-induced cell migration was hampered. The effect was more pronounced in cells with the highest syndecan-1 expression, indicating that syndecan-1 reduces cell migration in a dose-dependent manner. Similarly, none of the truncated constructs affected the basal level of cell migration (Figure 3A), but they all inhibited chemotactic migration along the FBS gradient (Figure 3B). Syndecan-1 constructs also decreased serum induced cell migration in STAV-AB transfectants (Figure 3D), and full-length syndecan-1 even decreased the basal level of cell migration (Figure 3C).

### Syndecan-1 inhibits cell motility and migration

**Figure 4:**
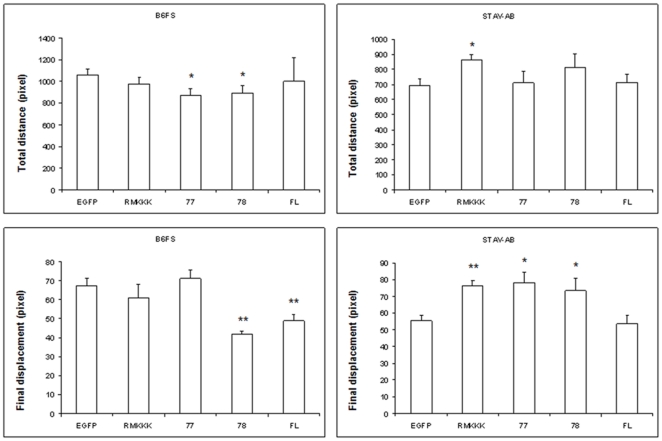
Effect of syndecan-1 overexpression on mesenchymal tumor cell motility measured by a random movement assay. B6FS and STAV-AB cells transfected with full-length syndecan-1 or various truncated variants were monitored using time-lapse video microscopy as described in Materials and Methods. Twenty individual cells from each cell line were selected and tracked. Total movement distances and final displacements were calculated. The reported values are Means ± SEM. Statistical significance: ^*^ *p*<0.05, ^**^ *p*<0.001, compared to EGFP control.

**Figure 5:**
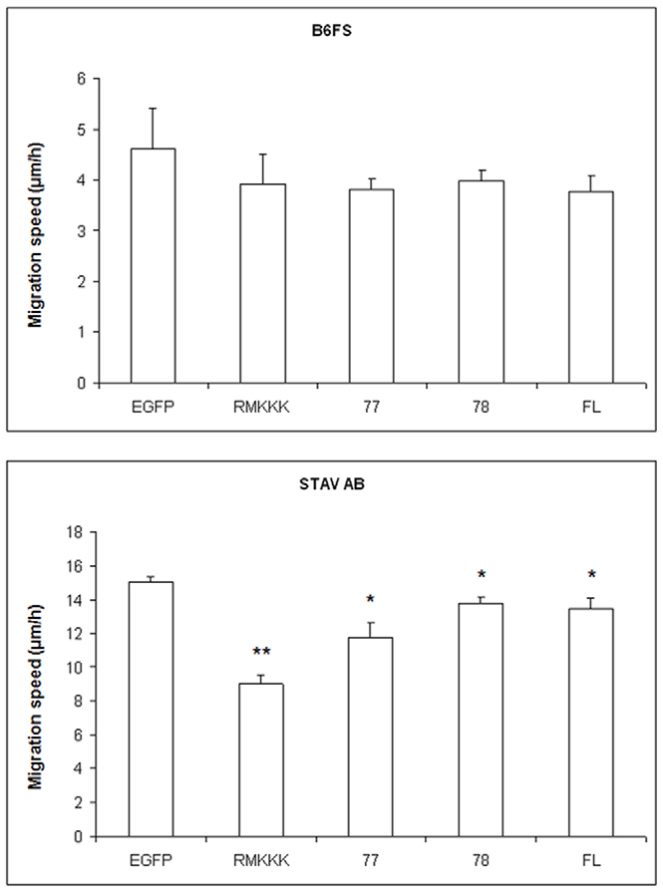
Effect of syndecan-1 overexpression on *mesenchymal* tumor cell motility/migration measured by a wound healing assay. B6FS and STAV-AB cells transfected with full-length syndecan-1 or various truncated variants were cultured in 60 mm petri-dishes until monolayer confluence was achieved. The cell layer was then wounded and the wound closure was monitored at various time points using a digital image processor connected to a microscope. Width measurements were taken across the wound at six different positions along it. Progression of the cell front was quantified and calculated as migration speed (µm/h). The assay was performed three times independently, with triplicate measurements in each. The reported values are Means ± SEM of 18 observations from 1 representative experiment. Statistical significance: ^*^ *p*<0.05, ^**^ *p*<0.001, compared to EGFP control.

In order to measure the changes in cell motility following syndecan-1 overexpression, the movement of individual cells was visualized using time-lapse video microscopy. All fibrosarcoma transfectants had a tendency for migrating shorter total distances, suggesting decreased level of cell motility. More interestingly, the full-length syndecan-1 and 78 transfectants showed significantly reduced final displacements, indicating that their cell movement was restricted to a smaller area. However, the STAV-AB MM cells showed a tendency of smaller final displacement only in the full-length and actually increased effects in the other transfectants (Figure 4 and Videos S1, S2, S3, S4, S5, S6, S7, S8, S9, and S10). Furthermore, when we examined the effect of syndecan-1 in a wound healing assay, both the full-length syndecan-1 and its truncated variants inhibited MM cell migration shown as slower wound closure, the RMKKK construct giving the most pronounced effect. As for the effect on fibrosarcoma cells, all transfectants displayed a trend of decreased ability to migrate (Figure 5).

### RMKKK deletion hampers the nuclear translocation of syndecan-1

**Figure 6:**
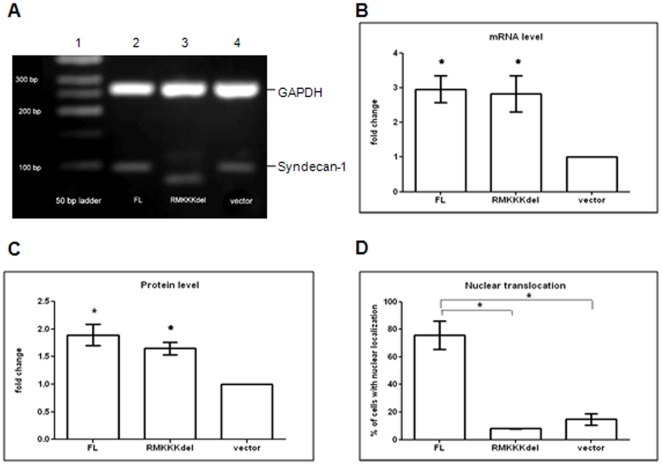
Syndecan-1 expression and subcellular distribution in B6FS cells transfected with full-length and RMKKK deleted constructs. A. Electrophoresis on agarose gel showed the expression of RMKKKdeleted construct in the transfected cells, giving a shorter amplimer(lane 3) than the full-length syndecan-1 product (lane 2) or the endogeneous syndecan-1 (lane 4). B. Upregulation of syndecan-1 mRNA in the transfected cells. Fold change was calculated based on the intensity of the bands normalized to the vector control, with GAPDH as reference gene. Results represent mean of 4 independent experiments. Expression of syndecan-1 protein (C) and the level of nuclear translocation (D) was examined by immunocytochemistry. Fold change in protein level was calculated by measuring the fluorescence intensity. Nuclear translocation of syndecan-1 was quantified by calculating the percent of cells showing nuclear immunoreactivity to the total number of cells. Results presented are Means ±SEM from at least 2 independent experiments. Statistical significance: ^*^ *p*<0.05, compared to vector control as indicated (D).

The deletion of the RMKKK sequence was verified by sequencing and by gel electrophoresis, yielding a shorter fragment in cells transfected with the RMKKK deleted construct compared to the full-length syndecan-1 (Figure 6A). Syndecan-1 mRNA levels, both in full-length and RMKKK deleted transfectants were approximately three fold increased compared to cells transfected with the corresponding vector (Figure 6B). Overexpression of syndecan-1 at protein level corresponded to 1.5 to two fold increase compared to cells transfected with the vector control (Figure 6C).

Overexpression of the RMKKK deleted construct resulted in considerable decrease of the nuclear syndecan-1 as compared to cells transfected with the wild type full-length syndecan-1. The intensity of nuclear reactivity was decreased two fold in cells transfected with the RMKKK deleted construct compared to cells transfected with full-length one (data not shown).

Simultaneously, the proportion of cells showing nuclear staining was 76% after transfection with full-length syndecan-1 compared to 15% in cells transfected with the empty vector and 8% in cells transfected with the RMKKK deleted mutant (Figure 6D).

### Syndecan-1 influences cell adhesion/migration/chemotaxis-related genes

**Figure 7:**
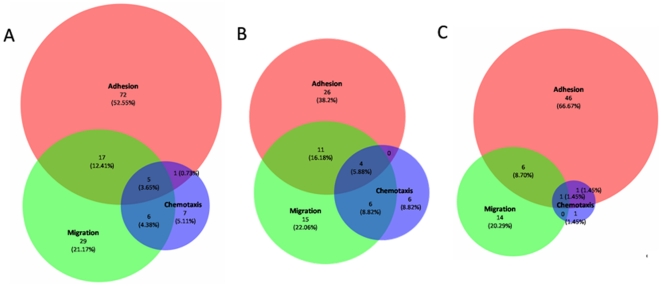
Affymetrix microarray for gene expression pattern influenced by syndecan-1 overexpression in MM STAV-AB cells. Venn diagram showing both distribution and proportion of genes connected to individual GO groups and the combinations of different GO groups (threshold is above 5% significance level and two fold changes). A. All regulated genes. B. upregulated genes. C. downregulated genes.

The performed bioassays show that the overexpression of syndecan-1 may influence mesenchymal tumor cell adhesion, and decrease the tumor cell motility and migration. To investigate the effects of syndecan-1 overexpression on general transcriptional modulation in STAV-AB cells, we performed microarray analysis, which showed a total of 2878 genes regulated by overepression of syndecan-1. Of these genes, 138 were identified to be adhesion, migration and chemotaxis related genes based on the Gene Ontology (GO) selection. We further clustered these terms into different combinations to show their overlapping functions (Figure 7A and Table S1). Among the total 138 genes, 71 genes were up-regulated (Figure 7B) and 67 genes were down-regulated (Figure 7C). The majority of these genes (53%) were associated with adhesion, compared to migration (21%) and chemotaxis (5%). The intersections of Venn diagram showed that many genes were involved in two or three of these functional categories. Thus, 17 genes were involved in both adhesion and migration, 6 genes were involved in both migration and chemotaxis, and 5 genes were associated with all three groups (Figure 7A).

When genes modulated by syndecan-1 overexpression were grouped according to the class of protein, some of these genes were associated with more than one of the above functional groups. Thus, for example, overexpression of syndecan-1 affected extracellular matrix molecules, growth factors and growth factor receptors, cytokines and chemokines, adhesion molecules, cytoplasmic proteins and cell motility-associated molecules (Table S1). Top two up-regulated genes were formyl peptide receptor 1 and metastasis suppressor 1 (MTSS1) and top two down-regulated genes were signaling lymphocyte activation molecule family member 7 (SLAMF7) and leucine rich repeat containing 7 (LRRC7). Many of the gene products bind to syndecans or heparan sulfate, and the data form the basis for further detailed investigation of syndecan-1 function.

## Discussion

Cell adhesion and migration are complex and inter-dependent cellular processes. Cell movement requires adhesion to and release from ECM, and excessive adhesion will inhibit cell motility. Many experimental techniques for analyzing cell motility also examine the directional movement, or migration, of cells in response to gradients of stimuli. These can be chemotaxis along a soluble, chemical gradient, haptotaxis along a gradient of adhesion sites for the underlying substrate or the ECM [31] or durotaxis in response to mechanical signals in the microenvironment [32]. Conceptually, directional cell migration has two sources: apart from the topography of the extracellular environment, there is also intrinsic cell directionality, including cell polarity and cell adhesion [33]. Cell adhesion can guide the directionality of migration by stabilizing lamellipodia [34], [35].

We have recently shown that both full-length syndecan-1 and its specific protein domains inhibit the proliferation of human MM and fibrosarcoma cells [22]. In the present study, we further demonstrate that syndecan-1 influences also the adhesion, motility and migration of these two mesenchymal tumor cell lines. Transfection of syndecan-1 into mesenchymal tumor cells that express very low levels of endogenous syndecan-1 is a way to influence intrinsic ability. We assume that cells stably overexpressing syndecan-1 acquire intrinsic cell directionality. It is important to note in this context that we recently demonstrated that overexpression of syndecan-1 simultaneously downregulates syndecan-2, as also verified in the present gene microarray analysis (data not shown). Overexpression of syndecan-1 also influences syndecan-4 expression, however, in a more variable manner as it is upregulated in MM cells but downregulated in fibrosarcoma cells [22]. It may thus be that the net effect of syndecan-1 transfection is only a minor increase in the total amount of syndecan-1, but a major change of the syndecan profile. This complex regulation must also be considered when evaluating effects of syndecan-1 overexpression on tumor cell behavior.

Full-length syndecan-1 enhanced fibrosarcoma cell adhesion, and the effect increased with high expression level. The weak expressers enhanced cell adhesion by 50%, whereas the strong ones increased by 150%. In contrast to cell adhesion, a chemotaxis assay showed that FBS-induced cell migration was inhibited in overexpressing cells in a dose-dependent manner. Interestingly, it was recently reported that overexpression of syndecan-2 enhanced the migration and invasion of melanoma cells [36]. It is also possible that the effects demonstrated in this study may partly be due to an indirect influence from altered syndecan-2 and syndecan-4 expressions induced by syndecan-1 transfection. A dose-dependent regulation of cell motility by syndecan-1 is also supported by the fact that in the absence of syndecan-2, syndecan-4 may govern cell movement according to a dose-related mechanism; both high and low levels of syndecan-4 diminish cell motility by alternatively providing a too tight or a too loose contact with the substratum [37], [38], [39]. All these observations imply that overlapping functions exist between members of the syndecan family [14], [40].

A similar syndecan-1-associated inhibition of fibrosarcoma cell motility was also seen with time-lapse microscopy, where the total distance moved was unaltered in the overexpressing cells, whereas the final displacement was decreased. This reduced migration distance with a preserved level of motility indicates that syndecan-1 overexpression restricted cell movement to a more limited area. Such restricted migration and decreased chemotaxis of syndecan-1 transfectants may thus correlate to the enhanced cell adhesion seen in overexpressing cells. When we examined the effect of syndecan-1 on the motility/migration of mesenchymal tumor cells using the wound-healing assay, the overexpressing cells showed slower wound closure. This effect may not only be a consequence of decreased cell migration but possibly also of the reduced cell proliferation as we have recently shown [22]. Influence of durotaxis from the wound can't be ignored when evaluating the net effect on cell migration as cells have a tendency to migrate into a region denuded of cells.

Overexpression of full-length syndecan thus affects both cell adhesion and migration, as two tightly-related cellular processes. The migratory effect may partly be secondary to the changes in adhesion, and the gene expression analysis shows that majority of regulated genes are adhesion related. However, there are many genes associated to migration and chemotaxis, and many of them have multiple functions.

MTSS1, one of the most up-regulated genes, is an actin and membrane binding protein. It has been demonstrated as a tumor suppressor and to be downregulated in bladder cancer, hepatocellular carcinoma and gastric cancer [41], [42], [43]. It has also been shown to suppress growth as well as invasive, migratory, and adhesive properties of a breast cancer cell line [44]. While the most down-regulated gene, SLAMF7 (CD319), is expressed on cytotoxic lymphoctes, activated B-cells, and mature dendritic cells [45], [46]. It has been shown that it mediates cytotoxicity of NK-cells [47], and induces proliferation and autocrine cytokine expression on human B lymphocytes [48]. The SLAM family of receptors plays roles in lymphocyte development, cytotoxicity, immunity, cell survival and cell adhesion [49]. The second down-regulated gene LRRC7, also known as Densin-180, is a transmembrane protein containing one PDZ domain. LRRC7 has been shown to interact with N-Cadherin [50] and may thus provide a mechanism for transendothelial migration in cancer cells. Hyaluronan- and proteoglycan link protein 1 (HAPLN1), which was also downregulated, has been shown to play a pro-tumorigenic role in malignant pleural mesothelioma [51]. These reports are in line with our present findings demonstrating that overexpression of syndecan-1 negatively regulated MM cell migration. Integrin alpha5 (ITGA5) and integrin beta1 (ITGB1) were upregulated whereas integrin beta5 was downregulated, thus supporting the notion of close cooperation between syndecans and integrins. Together, they regulate the global gene expression and a crosstalk of signaling pathways. The varying effects of syndecan-1 overexpression on cell behavior comparing the two cell lines must be put into this context.

In an attempt to dissect the contribution of the different functional protein domains of this PG, the two mesenchymal tumor cell lines were transfected with three truncated variants of syndecan-1. The effects on enhanced cell adhesion seem mainly to depend upon the EC domain with its GAG chains, as in its absence cell adhesion is not enhanced. Indeed the two shorter constructs with no EC domain, i.e. 77/EGFP and RMKKK/EGFP, actually inhibited cell adhesion. Interestingly, the 78/EGFP construct, which contains the short DRKE motif remnant of the EC domain (mimicking the structure that remains in the plasma membrane after syndecan-1 shedding) also enhanced adhesion to some extent. The different effects seen when comparing the 77/EGFP and 78/EGFP constructs, which only differ in the presence of the four extra amino acid residues in the latter, are intriguing. It may indicate a biological significance for the juxtamembrane DRKE motif, presumably on the oligomerization of the syndecan-1. The finding that the 78/EGFP construct, but not the 77/EGFP constructs also decreased the final displacement in the cell random movement assay, in a similar way to full-length syndecan-1, also supports the idea that reduced migration may correlate with increased adhesion.

Although none of the truncated constructs had any effect on the basal level of fibrosarcoma cell migration in the Transwell assay, they all inhibited the chemotactic migration along a FBS gradient. Thus, this kind of migration seems to depend more on the cytoplasmic portion of the PG rather than on its EC domain and attached GAGs. All the truncated constructs also inhibited wound closure of the two mesenchymal tumor cells. This was seen even with the short RMKKK/EGFP construct, which had the most pronounced effect on MM cells. It therefore seems that the syndecan-1 effect on cell migration is not only dependent on its cell surface interaction, but also related to its presence in the nucleus. The function of nuclear syndecan-1 is still obscure. Deletion of the RMKKK motif abolished the nuclear translocation of syndecan-1. This RMKKK motif binds to cytoskeleton components and it may in this way influence cell adhesion-related function. Moreover, it acts as a NLS for syndecan-1, and transfection of the RMKKK/EGFP construct results in a nuclear accumulation of the transcript, suggesting the existence of nuclear ligands to the motif [21]. The finding that heparanase decreases the amount of nuclear syndecan-1 indicates that syndecan-1 in the nucleus can also have other nuclear binding sites [19]. It can be speculated that the free RMKKK fragment blocks the translocation of native syndecan-1 into the nucleus, and this could be one way to hamper the function of nuclear syndecan-1, resulting in transcriptional regulation. The effects seen with this motif unique to the syndecans, indicate that the function of syndecans on the migratory capacity is also influenced by events in the nucleus.

A study of the differential roles for membrane-bound and soluble syndecan-1 in breast cancer progression has recently been published. Proteolytic conversion of syndecan-1 from a membrane-bound into a soluble molecule marks a switch from a proliferative to an invasive phenotype [20]. Increased invasiveness was observed in another fibrosarcoma cell line HT-1080 when transfected with the same full-length and 78 truncated syndecan-1 constructs as we used, and local growth was faster in the full-length syndecan-1 than the 78 transfectants. It is presumed that the whole protein and maybe the shedding is needed for the local development of the tumor, but the intracellular and transmembrane domain is sufficient to promote the formation of metastasis [52]. We found that the full-length syndecan-1 and the remnants of syndecan-1 after shedding (the 78/EGFP construct), negatively regulated fibrosarcoma B6FS cell migration, and this can be partly due to the different phenotypes of these two fibrosarcoma cell lines. We also found that the 78/EGFP construct displayed opposite effects on cell motility in terms of final displacement between fibrosarcoma and MM cell lines, showing the cell type-specific effects. Further investigations on syndecan-1 interacting ligands and signaling pathways are on-going in our laboratory.

Taken together, our results show that syndecan-1 decreases migration and motility, and enhances adhesion of mesenchymal tumor cells in an expression level-dependent manner. Distinct protein domains have different effects: the extracellular domain is more important for promoting cell adhesion, while the transmembrane and cytoplasmic domains are sufficient for inhibition of cell migration; cell adhesion and migration seem to depend also on the nuclear localization of syndecans. Our study demonstrates that syndecan-1 plays an important role in mesenchymal tumor behavior. Moreover, our study provides new inputs into the better understanding of structure-function relationship of this PG in tumor progression.

## Materials and Methods

### Cell lines and their culture

STAV-AB human MM cells were grown in RPMI 1640 medium containing 25 mM HEPES (42401, Gibco, Grand Island, NY, USA) and 2 mM L-Glutamine, supplemented with 10% human AB serum (growth medium) [53]. B6FS human fibrosarcoma cells were grown in RPMI 1640+ glutaMAX™-I (72400, Gibco) supplemented with 10% foetal bovine serum (FBS) and Gentamicin 50 µg/ml (Gibco) (growth medium) [54]. All cells were cultured in 75 cm^2^ Tissue Culture Flasks (Sarstedt, Newton, NC, USA), in humidified 5% (v/v) CO_2_ at 37°C and culture medium was changed twice a week. Both cell lines are of mesenchymal origin and were selected based on their low endogenous expression levels of syndecan-1 [22], [55].

### Plasmids and generation of stably-transfected cell lines

The plasmids and subsequent stable transfection of cells were described in detail in our previous publication [22]. The 78/EGFP, 77/EGFP and RMKKK/EGFP constructs are three truncated variants of the human full-length syndecan-1/EGFP construct. The 78/EGFP lacks the extracellular domain with the exception of the juxtamembrane DRKE sequence, the 77/EGFP lacks the entire extracellular domain, and the RMKKK/EGFP contains only the nuclear localization signal (NLS). The pEGFP-N1 vector was used as a negative control.

MM and fibrosarcoma cells were transfected with the constructs above, using Effectene Transfection Reagent (Qiagen GmbH, Hilden, Germany). Optimization of the transfection was carried out according to the manufacturer's guidelines. To obtain stable transfectants, the EGFP positive cells were selected by Geneticin incubation (G418, Roche Diagnostics GmbH, Mannheim, Germany). Mock-transfected cells were used as a reference for selection. In this study, only the stably-transfected cell lines were used and cultured under geneticin pressure (200 µg/mL) B6FS stable tansfectantsof the full-length syndecan-1/EGFP and the RMKKK/EGFP were further separated based on their EGFP intensity by using fluorescence-activated cell sorting (FACS, BD FACSAria™, BD Biosciences, San Jose, California, USA). The FACS was performed by the Flow Cytometry Unit at Karolinska University Hospital Huddinge, Sweden). The subpopulations with high or low EGFP intensity were isolated and subcultured as strong or weak expressers respectively.

### Generation of the RMKKK deletion construct

In order to study the importance of the RMKKK sequence as a NLS, we designed a new construct that lacks the RMKKK sequence from a plasmid carrying the gene encoding the full-length syndecan-1 (pN1-flsyn1). The pN1-flsyn1 plasmid was constructed by deleting the EGFP gene from the commercially available pN1-EGFP plasmid (BD Biosciences, Clontech, Palo Alto, CA, USA) by digestion with EcoRI and NotI restriction enzymes and replacing it with the gene encoding the full-length syndecan-1. In order to construct the RMKKK deletion plasmid, we designed a primer pair flanking the RMKKK region (FwRMKKKdel: gacgaaggcagctactccttggag; RevRMKKKdel: gtacagcatgaaacccaccaggca) in the syndecan-1 gene. We amplified the pN1-flsyn1 plasmid by PCR reaction using a Phusion® Hot Start DNA Polymerase (Phusion Site-directed Mutagenesis Kit, Finnzymes, Espoo, Finland). Both primers were phosphorylated at the 5′ end to allow direct ligation after the PCR. The PCR reaction was carried out in a final volume of 50 µl. The reaction mixture contained 10 µl of 5× Phusion HF Buffer, 1 µl of 10 mMdNTPs, 50 pmol of both primers, 200 ng template DNA and 1 U of Phusion® Hot Start DNA Polymerase. The amplification was done using a two-step cycling protocol: denaturation at 98°C for 30 sec, annealing and elongation at 72°C for 80 sec, followed by a final incubation at 72°C for 5 min. The resulted linearized DNA represents the pN1flsyn1_RMKKK*del* plasmid (RMKKK-del), lacking the RMKKK sequence. The full-length syndecan-1 sequence was deleted from the same pN1-flsyn1 construct. Briefly, a primer pair flanking the full-length syndecan-1 gene was designed (Fwsyn1del: agcggccgcgactctagatcataat; Revsyn1del: aagcttgagctcgagatctgagtcc). The PCR product amplified with the aid of Site-directed Mutagenesis Kit (Finnzymes) was the pN1-empty plasmid (vector), and used as negative control.

25 ng of the PCR products were recircularized using Quick T4 DNA ligase (provided with the Phusion Site-directed Mutagenesis Kit, Finnzymes) in a reaction at 25°C for 5 minutes. Chemically competent *E. coli* cells were transformed with these plasmids; the cells were plated on LB-agar plates containing 50 µg/mL kanamycin as a selection agent. Plasmid DNA was isolated using QiAmpMiniprep spin kit (Qiagen GmbH, Hilden, Germany), as suggested by the manufacturer. These newly designed constructs were sequenced to verify if the deletion was correct and complete. The sequencing was performed by CybergeneAB, Stockholm, Sweden.

### Reverse transcription polymerase chain reaction (RT-PCR)

Total cellular RNA was isolated from subconfluent B6FS cells, using the High Pure RNA Isolation Kit (Roche Diagnostics GmbH Mannheim, Germany). The yield and purity of RNA preparations were estimated spectrophotometrically by measuring the absorbance at 260 nm, and the A260/A230 and A260/A280 ratio, respectively. From each sample, 2 µg of RNA was reverse transcribed and the cDNAs were amplified using the First Strand cDNA Synthesis Kit (Pharmacia Biotech, Uppsala, Sweden). A region of 94 base-pair containing the RMKKK sequence was amplified using the following primers in the vicinity of the RMKKK region: fwRMKKKdel2: ggctcatctttgctgtgtgc; revRMKKKdel2: gcttgtttcggctcctccaa. The product of RMKKK deleted syndecan-1 (79 bp) can be distinguished from the product of native full-length syndecan-1, which is longer (94 bp). For the PCR reaction, 3 µl of the RT reaction was used. The reaction mixture contained 5 µl 5× Phusion HF Buffer, 0,5 µl of 10 mMdNTPs, 25 pmol of both primers, and 0,5 U of Phusion® Hot Start DNA Polymerase (Finnzymes) in a final volume of 25 µl. To allow the semiquantitative analysis of the sequences, we also added to the reaction 25 pmol of a primer pair for glyceraldehyde-3- phosphatase dehydrogenase(GAPDH) (GAPDH-se: acatcatccctgcctctactgg, GAPDHas: agtgggtgtcgctgttgaagtc) [55], which resulted in a 214 bp product. Amplification of the cDNA product was done for 25 cycles using the following parameters: denaturation at 98°C for 30 sec, annealing at 67°C for 30 sec and elongation at 72°C for 1 min, followed by a final incubation at 72°C for 5 min. RT-PCR products were analyzed on 3% agarose gels. Semiquantitative measurement of mRNA expression was carried out with the FluorChem Imaging System and AlphaEase FC Software V5.0.0 (Alpha Innotech Inc.) using GAPDH as a reference gene.

### Detection and subcellular localization of newly synthesized syndecan-1

Expression and distribution of syndecan-1 proteins was further examined using immunocytochemical analysis and fluorescent microscopy. Stably transfected cells were seeded on to POLYSINE coated microscopy slides (Menzel-Gläser, Braunschweig, Germany) and allowed to adhere for 48 h before fixation in 3% paraformaldehyde. After permeabilization with 0.1% Triton X-100 (Sigma, Steinheim, Germany) non-specific binding was blocked with 3% goat serum (Dako A/S, Glostrup, Denmark) for 30 min. Incubation with the primary antibody diluted 1∶4, (Mouse anti Human CD138 monoclonal antibody (MCA-681) Serotec LTD, Kidlington, Oxford, England) or with mouse IgG1 (Dako A/S, Glostrup, Denmark)as negative control was performed overnight at 4°C, followed by 30 min incubation with the secondary antibody (Alexa 488 goat anti-mouse F(ab′)2 fragment of IgG (H+L), (Molecular Probes, Leiden, The Netherlands, A11017) in the dark at room temperature. Samples were then counterstained with 1 mg/L bisbenzimide H33342 (Fluka, Steinheim, Germany). Detailed visualization was performed using Nikon microphot-FXA EPI-FL3 fluorescence microscope. Images were processed using ImageJ 1.43 software allowing measurement of fluorescence intensity. For expression of syndecan-1 protein, background was subtracted, and fluorescence intensity of the cells was measured in 7 randomly selected visual fields for each slide. For quantification of nuclear syndecan-1, the intensity of nuclear fluorescence was measured in 50 randomly selected cell nuclei for each transfectant. We also calculated the proportion of cells showing nuclear staining to total number of cells in the same visual field, 100–150 cells were evaluated for each sample.

### Adhesion assay

B6FS syndecan-1 overexpressing cells were seeded at 5.000 cells per well in a 96-well plate (Corning Incorporated, Corning, NY, USA), in its growth medium containing geneticin. After 5 minutes, the wells were washed twice with the culture medium to remove the floating cells. Adherent cells were lysed using Cell Lysis Buffer (Cat. 90130, Chemicon, Billerica, MA, USA) and the cell numbers were quantified using CyQUANTGR® Dye (Cat. 90132, Chemicon) as measured with a fluorometer (BioTek Instruments, Winooski, VT, USA) with a 480/520 nm filter set. This assay was performed in triplicate.

### Chemotaxis assay

Directional migration of B6FS syndecan-1 overexpressing cells was assessed using a 24-Transwell plate (Cat. 3422, Corning Incorporated). After 24 h serum starvation, 50.000 cells were seeded into an upper well of the chamber in serum-free medium. FBS (10%), as a chemoattractant was placed in the bottom chamber. After 6 h the cells that had migrated through the microporous membrane were dislodged from the outer surface of the insert using Trypsin 0.5% EDTA (Gibco), and then lysed using Cell Lysis Buffer (Cat. 90130, Chemicon). The cell numbers were quantified as described above for the cell adhesion assay. This assay was performed in triplicate.

### Random movement assay

STAV-AB and B6FS syndecan-1 overexpressing cells were seeded in a 12-well glass bottom culture plate (MatTek Corporation, Ashland, MA, USA), and incubated at 37°C for 6 hours before transfer to a Leica DMIRE2 Inverted Laboratory Microscope (McBain Instruments, Simi Valley, CA,USA) for imaging. Cells were maintained during imaging in normal culture condition. Five observation fields were randomly selected and time-lapse imaging was performed every 15 minutes over 16 hours with a 10× dry objective. This yielded a video of 65 photos for each single cell monitored. The movements of 20 individual cells for each cell line were tracked using ImageJ software (NIH). Total distances and final displacements of moving cells were calculated based on path tracking data as pixels.

### Wound healing assay

STAV-AB and B6FS syndecan-1 overexpressing cells were seeded in 60 mm petri-dishes and cultured to confluence in their growth media containing geneticin. The cell monolayer was then wounded by scratching with a sterile 200 µl pipette tip. Detached cells were removed by washing twice with culture medium. Subsequent wound closure was monitored at 3, 6 and 12 hours using a digital image processor connected to a microscope. Width measurements were taken across the gap at six different positions along the wound. Cell motility was quantified by image analysis (ImageJ 1.4.3.67 Launcher Symmetry Software, NIH, Bethesda, MD, USA) and calculated as migration speed (µm/h). This assay was performed three times independently with triplicates in each.

### RNA isolation and microarray analysis

The STAV-AB MM cells stably overexpressing full-length syndecan-1, or the EGFP vector were cultured for about 36 hours. Total RNA was isolated from sub-confluent cell cultures, using the High Pure RNA Isolation Kit (Roche, Mannheim, Germany), with optional on-column DNase 1 digestion, according to the supplied protocol. The yield and purity of the RNA preparations were estimated spectrophotometrically by measuring the UV absorbance at 260 nm and calculating the A260/A280 ratio, respectively. Three independent experiments were performed.

The above RNA samples were applied to gene microarray analysis using GeneChip® Human Gene 1.0 ST Arrays (Affymetrix, Inc., Santa Clara, CA, USA), representing more than 22.000 well-annotated human genes. Target synthesis and hybridizations were performed in the Affymetrix core facility (NOVUM, Karolinska Institutet, Huddinge, Sweden) according to standard protocols. Affymetrix Expression Console (http://www.affymetrix.com/products_services/software/specific/expression_console_software.affx) was used for the data preprocessing, with the PLIER method for summarization, PM-GCBG for background correction and Global Median for normalization. Expression levels in the different groups were compared using Student's t-tests, and, as a measure of corresponding false discovery rates, q-values were calculated using the q-value package in R (http://www.rproject.org/). All data is MIAME compliant and the raw data has been deposited in the MIAME compliant database Gene Expression Omnibus (the accession number GSE21401, http://www.ncbi.nlm.nih.gov/geo/info/linking.html). By applying a filter of at least a two-fold change in mean expression level with P<0.05 for significance, we screened for modulated genes in the full-length syndecan-1 overexpressing cells with the EGFP mock-transfectants as reference. Gene Ontology (GO) (http://amigo.geneontology.org/cgi-bin/amigo/go.cgi) was used to select genes related to cell adhesion/migration/chemotaxis from the total regulated genes based on their GO annotations. Venn diagrams were constructed for analysis of these GO terms using a web-based application [56].

### Statistical analysis

Statistical significance was evaluated using the Student's *t*-test and the one way completely randomized variance analysis (ANOVA) using the Microcal Origin (version 5.0) software. The null hypothesis of no difference was rejected at α = 0.05.

## Supporting Information

Table S1 Genes modulated by syndecan-1 overexpression in MM STAV-AB cells. All genes are selected with at least a 95% confidence interval and filtered with two fold changes. The table is sectioned according to GO terms: adhesion, migration and chemotaxis; as well as their respective combinations. Figure 7 was generated based on the data in this table.
(0.28 MB DOC)

Video S1 Videos S1-S10 for Figure 4. Random movement assay on the syndecan-1 overexpressing cells. The cells were seeded in a 12-well glass bottom culture plate and incubated at 37°C for 6 hours before transfer to a Leica DMIRE2 Inverted Laboratory Microscope for imaging. Cells were maintained during imaging in normal culture condition. Five observation fields were randomly selected and time-lapse imaging was performed every 15 minutes over 16 hours with a 10× dry objective. Thus a video of 65 photos for each single cell monitored was yielded and can be played using Quicktime player or Windows Media player software. A representative video of each transfectant was selected to show its random movement: Video S1, B6FS EGFP; Video S2, B6FS RMKKK/EGFP; Video S3, B6FS 77/EGFP; Video S4, B6FS 78/EGFP; Video S5, B6FS FL/EGFP. Video S6, STAV-AB EGFP; Video S7, STAV-AB RMKKK/EGFP; Video S8, STAV-AB 77/EGFP; Video S9, STAV-AB 78/EGFP; Video S10, STAV-AB FL/EGFP. The difference of motility between different transfectants can be seen by comparison of videos of respective transfectants.
(6.93 MB AVI)

Video S2 (4.82 MB AVI)

Video S3 (4.34 MB AVI)

Video S4 (3.79 MB AVI)

Video S5 (4.56 MB AVI)

Video S6 (3.59 MB AVI)

Video S7 (3.19 MB AVI)

Video S8 (3.10 MB AVI)

Video S9 (5.09 MB AVI)

Video S10 (3.84 MB AVI)
