# Supplementary material for: Specific Syndecan-1 Domains Regulate Mesenchymal Tumor Cell Adhesion, Motility and Migration
Source: PLoS One. 2011 Jun 23;6(6):e14816. doi: 10.1371/journal.pone.0014816 (PMC3121713; doi:10.1371/journal.pone.0014816)
Supplement: Table S1 — Genes modulated by syndecan-1 overexpression in MM STAV-AB cells. All genes are selected with at least a 95% confidence interval and filtered with two fold changes. The table is sectioned according to GO terms: adhesion, migration and chemotaxis; as well as their respective combinations. Figure 7 was generated based on the data in this table. (0.28 MB DOC) [file pone.0014816.s001.doc]

**Supporting Information**

**Supplementary Table 1. Genes modulated by syndecan-1 overexpression in MM STAV-AB cells**

| GO term/  Gene ID | | | | Gene product | F.C. | p-value | q-value |
| --- | --- | --- | --- | --- | --- | --- | --- |
| Adhesion (72) | | | | | | | |
|  | MTSS1 | | | Metastasis suppressor 1 | 17,25 | 2E-05 | 0,0043 |
|  | VNN1 | | | Vanin 1 | 16,35 | 1E-05 | 0,0032 |
|  | DPP4 | | | Dipeptidyl-peptidase 4 (CD26, adenosine deaminase complexing protein 2) | 12,65 | 4E-08 | 0,0003 |
|  | POSTN | | | Periostin, osteoblast specific factor | 9,52 | 8E-06 | 0,0029 |
|  | COL8A1 | | | Collagen, type VIII, alpha 1 | 7,88 | 0,007 | 0,0599 |
|  | TNXA | | | Tenascin XA pseudogene | 6,99 | 0,0022 | 0,0335 |
|  | TNXB | | | Tenascin XB | 6,93 | 0,0018 | 0,0295 |
|  | SIRPA | | | Signal-regulatory protein alpha | 6,82 | 2E-05 | 0,0045 |
|  | CDON | | | Cdon homolog (mouse) | 6,48 | 4E-06 | 0,0022 |
|  | DSC3 | | | Desmocollin 3 | 6,11 | 1E-05 | 0,0037 |
|  | LPXN | | | Leupaxin | 5,74 | 0,0004 | 0,0144 |
|  | CNTN4 | | | Contactin 4 | 5,61 | 0,0002 | 0,0104 |
|  | DDR2 | | | Discoidin domain receptor tyrosine kinase 2 | 5,52 | 6E-06 | 0,0026 |
|  | GNE | | | Glucosamine (UDP-N-acetyl)-2-epimerase/N-acetylmannosamine kinase | 5,08 | 0,0003 | 0,0127 |
|  | CAV1 | | | Caveolin 1, caveolae protein, 22kDa | 4,19 | 0,0119 | 0,0814 |
|  | LIMA1 | | | LIM domain and actin binding 1 | 3,97 | 0,0005 | 0,0169 |
|  | SCARB2 | | | Scavenger receptor class B, member 2 | 3,14 | 8E-06 | 0,0030 |
|  | ATP2C1 | | | ATPase, Ca++ transporting, type 2C, member 1 | 3,04 | 0,0001 | 0,0084 |
|  | PTPRM | | | Protein tyrosine phosphatase, receptor type, M | 2,91 | 2E-05 | 0,0042 |
|  | SVEP1 | | | Sushi, von Willebrand factor type A, EGF and pentraxin domain containing 1 | 2,88 | 0,0002 | 0,0098 |
|  | SEMA5A | | | Semaphorin 5A | 2,68 | 6E-05 | 0,0063 |
|  | GPNMB | | | Glycoprotein (transmembrane) nmb | 2,28 | 0,0007 | 0,0195 |
|  | CREBL1 | | | cAMP responsive element binding protein-like 1 | 2,20 | 0,0002 | 0,0106 |
|  | FEZ1 | | | Fasciculation and elongation protein zeta 1 (zygin I) | 2,13 | 0,0026 | 0,0357 |
|  | FLRT2 | | | Fibronectin leucine rich transmembrane protein 2 | 2,09 | 0,0001 | 0,0084 |
|  | STAT5A | | | Signal transducer and activator of transcription 5A | 2,03 | 0,0008 | 0,0198 |
|  | CYFIP2 | | | Cytoplasmic FMR1 interacting protein 2 | -2,08 | 0,0037 | 0,0428 |
|  | CDH6 | | | Cadherin 6, type 2, K-cadherin (fetal kidney) | -2,13 | 0,0254 | 0,1236 |
|  | THRA | | | Thyroid hormone receptor, alpha (erythroblastic leukemia viral (v-erb-a) oncogene homolog, avian) | -2,18 | 0,0002 | 0,0098 |
|  | KITLG | | | KIT ligand | -2,18 | 0,0158 | 0,0953 |
|  | L1CAM | | | L1 cell adhesion molecule | -2,19 | 0,0089 | 0,0690 |
|  | PCDHB4 | | | Protocadherin beta 4 | -2,20 | 0,0407 | 0,1609 |
|  | MAGI1 | | | Membrane associated guanylate kinase, WW and PDZ domain containing 1 | -2,21 | 0,0055 | 0,0535 |
|  | PCDHB7 | | | Protocadherin beta 7 | -2,23 | 0,026 | 0,1253 |
|  | ITGB5 | | | Integrin, beta 5 | -2,27 | 0,0122 | 0,0827 |
|  | TGFBI | | | transforming growth factor, beta-induced, 68kDa | -2,29 | 0,0224 | 0,1155 |
|  | PCDHB16 | | | Protocadherin beta 16 | -2,36 | 0,0061 | 0,0562 |
|  | PPFIBP1 | | | PTPRF interacting protein, binding protein 1 (liprin beta 1) | -2,45 | 0,0018 | 0,0296 |
|  | COL18A1 | | | Collagen, type XVIII, alpha 1 | -2,53 | 0,0006 | 0,0176 |
|  | CDH1 | | | Cadherin 1, type 1, E-cadherin (epithelial) | -2,69 | 0,002 | 0,0318 |
|  | FLNB | | | Filamin B, beta (actin binding protein 278) | -2,71 | 0,0013 | 0,0256 |
|  | APLP1 | | | Amyloid beta (A4) precursor-like protein 1 | -2,84 | 0,0027 | 0,0368 |
|  | PCDHB17 | | | Protocadherin beta 17 pseudogene | -2,88 | 0,0208 | 0,1106 |
|  | COL3A1 | | | Collagen, type III, alpha 1 (Ehlers-Danlos syndrome type IV, autosomal dominant) | -2,98 | 0,0013 | 0,0259 |
|  | CDK6 | | | Cyclin-dependent kinase 6 | -3,08 | 0,0013 | 0,0259 |
|  | THBS3 | | | Thrombospondin 3 | -3,09 | 0,0026 | 0,0357 |
|  | CLDN1 | | | Claudin 1 | -3,12 | 0,0017 | 0,0287 |
|  | JUP | | | Junction plakoglobin | -3,30 | 2E-05 | 0,0041 |
|  | ITGB6 | | | Integrin, beta 6 | -3,37 | 0,0113 | 0,0788 |
|  | NCAM1 | | | Neural cell adhesion molecule 1 | -3,42 | 0,013 | 0,0855 |
|  | CADM1 | | | Cell adhesion molecule 1 | -3,47 | 0,0002 | 0,0108 |
|  | PCDHB13 | | | Protocadherin beta 13 | -3,50 | 0,0014 | 0,0265 |
|  | PCDHB2 | | | Protocadherin beta 2 | -3,52 | 0,0007 | 0,0191 |
|  | PCDHB12 | | | Protocadherin beta 12 | -3,54 | 0,0091 | 0,0700 |
|  | PCDHB3 | | | Protocadherin beta 3 | -3,89 | 0,0062 | 0,0566 |
|  | AMIGO2 | | | Adhesion molecule with Ig-like domain 2 | -4,12 | 0,0202 | 0,1087 |
|  | PCDHB11 | | | Protocadherin beta 11 | -4,13 | 0,0005 | 0,0170 |
|  | NRXN3 | | | Neurexin 3 | -4,65 | 0,0022 | 0,0334 |
|  | FZD3 | | | Frizzled homolog 3 (Drosophila) | -4,80 | 0,0034 | 0,0409 |
|  | PCDHB8 | | | Protocadherin beta 8 | -4,90 | 0,0013 | 0,0256 |
|  | FLRT3 | | | Fibronectin leucine rich transmembrane protein 3 | -4,93 | 2E-05 | 0,0042 |
|  | PCDHB9 | | | Protocadherin beta 9 | -4,97 | 0,0013 | 0,0259 |
|  | PCDHB10 | | | Protocadherin beta 10 | -5,13 | 0,0045 | 0,0475 |
|  | HAPLN1 | | | Hyaluronan and proteoglycan link protein 1 | -5,13 | 0,0435 | 0,1672 |
|  | PCDHB6 | | | Protocadherin beta 6 | -5,16 | 0,0007 | 0,0193 |
|  | CDH3 | | | Cadherin 3, type 1, P-cadherin (placental) | -5,92 | 0,0016 | 0,0281 |
|  | PCDHB14 | | | Protocadherin beta 14 | -5,96 | 5E-05 | 0,0055 |
|  | ADAM23 | | | ADAM metallopeptidase domain 23 | -6,69 | 0,0002 | 0,0094 |
|  | MUC16 | | | Mucin 16, cell surface associated | -9,53 | 4E-05 | 0,0048 |
|  | DNAJC15 | | | DnaJ (Hsp40) homolog, subfamily C, member 15 | -20,89 | 0,0011 | 0,0237 |
|  | LRRC7 | | | Leucine rich repeat containing 7 | -33,39 | 0,0007 | 0,0195 |
|  | SLAMF7 | | | SLAM family member 7 | -49,36 | 6E-05 | 0,0061 |
| Migration (29) | | | | | | | |
|  | | PDGFRA | | Platelet-derived growth factor receptor, alpha polypeptide | 11,18 | 0,00359 | 0,0423 |
|  |  | PSG7 | | Pregnancy specific beta-1-glycoprotein 7 | 3,97 | 7,6E-05 | 0,0067 |
|  |  | ETS1 | | V-ets erythroblastosis virus E26 oncogene homolog 1 (avian) | 3,85 | 0,00014 | 0,0091 |
|  |  | PARD6B | | Par-6 partitioning defective 6 homolog beta (C. elegans) | 3,67 | 8,6E-06 | 0,0031 |
|  |  | NOX1 | | NADPH oxidase 1 | 3,61 | 2,3E-05 | 0,0000 |
|  |  | PDGFRB | | Platelet-derived growth factor receptor, beta polypeptide | 3,49 | 9,4E-05 | 0,0073 |
|  |  | DNER | | Delta/notch-like EGF repeat containing | 3,35 | 0,00023 | 0,0000 |
|  |  | VEGFC | | Vascular endothelial growth factor C | 3,34 | 0,00054 | 0,0170 |
|  |  | DCLK1 | | Doublecortin-like kinase 1 | 2,62 | 5,3E-06 | 0,0026 |
|  |  | SDCBP | | Syndecan binding protein (syntenin) | 2,45 | 2,3E-05 | 0,0000 |
|  |  | SERPINE2 | | Serpin peptidase inhibitor, clade E (nexin, plasminogen Activator inhibitor type 1), member 2 | 2,43 | 0,00275 | 0,0370 |
|  |  | SRGAP1 | | SLIT-ROBO Rho GTPase activating protein 1 | 2,34 | 0,00058 | 0,0175 |
|  |  | CCDC88A | | Coiled-coil domain containing 88A | 2,25 | 0,0003 | 0,0000 |
|  |  | SP100 | | SP100 nuclear antigen | 2,14 | 2,4E-05 | 0,0045 |
|  |  | CAV2 | | Caveolin 2 | 2,05 | 0,00135 | 0,0000 |
|  |  | TMSB4X | | Thymosin beta 4, X-linked | -2,01 | 0,00012 | 0,0084 |
|  |  | IGFBP5 | | Insulin-like growth factor binding protein 5 | -2,02 | 0,00514 | 0,0516 |
|  |  | PODXL | | Podocalyxin-like | -2,12 | 0,00091 | 0,0000 |
|  |  | BTG1 | | B-cell translocation gene 1, anti-proliferative | -2,12 | 0,00089 | 0,0000 |
|  |  | IRS2 | | Insulin receptor substrate 2 | -2,35 | 0,00022 | 0,0108 |
|  |  | AMOT | | Angiomotin | -2,37 | 0,01025 | 0,0000 |
|  |  | HMGCR | | 3-hydroxy-3-methylglutaryl-Coenzyme A reductase | -2,75 | 0,00038 | 0,0147 |
|  |  | MAP3K1 | | Mitogen-activated protein kinase kinase kinase 1 | -2,85 | 0,00012 | 0,0084 |
|  |  | IGF1R | | Insulin-like growth factor 1 receptor | -2,94 | 0,00077 | 0,0000 |
|  |  | CTGF | | Connective tissue growth factor | -3,43 | 0,00095 | 0,0000 |
|  |  | NR4A2 | | Nuclear receptor subfamily 4, group A, member 2 | -3,58 | 0,00025 | 0,0114 |
|  |  | HBEGF | | Heparin-binding EGF-like growth factor | -4,25 | 0,00018 | 0,0100 |
|  |  | TGFBR1 | | Transforming growth factor, beta receptor I (activin A receptor type II-like kinase, 53kDa) | -4,51 | 0,00122 | 0,0250 |
|  |  | F2R | | Coagulation factor II (thrombin) receptor | -6,49 | 0,00055 | 0,0170 |
| Chemotaxis (7) | | | | | | | |
|  | | | FPR1 | Formyl peptide receptor 1 | 38,26 | 8,9E-09 | 0,0001 |
|  |  |  | HLA-DRB3 | Major histocompatibility complex, class II, DR beta 3 | 10,65 | 7,1E-05 | 0,0065 |
|  |  |  | FPR3 | formyl peptide receptor 3 | 6,24 | 0,00035 | 0,0141 |
|  |  |  | FOSL1 | FOS-like antigen 1 | 4,82 | 0,00134 | 0,0260 |
|  |  |  | C3AR1 | Complement component 3a receptor 1 | 4,24 | 0,00019 | 0,0104 |
|  |  |  | CREB3 | cAMP responsive element binding protein 3 | 2,28 | 0,00749 | 0,0626 |
|  |  |  | C5 | Complement component 5 | -2,46 | 0,031 | 0,1391 |
| Overlap/Gene ID | | | | Gene product | F.C. | p-value | q-value |
| Adhesion, migration, chemotaxis (5) | | | | | | |  |
|  | | | NRP2 | Neuropilin 2 (NRP2) | 15,35 | 7,13E-05 | 0,0065 |
|  |  |  | IL8 | Interleukin 8 (IL8) | 9,94 | 0,0005 | 0,0168 |
|  |  |  | SAA2 | Serum amyloid A2 (SAA2) | 2,09 | 0,022 | 0,1148 |
|  |  |  | SAA1 | Serum amyloid A1 (SAA1) | 2,04 | 0,011 | 0,0784 |
|  |  |  | TGFB2 | Transforming growth factor, beta 2 (TGFB2) | -13,51 | 0,0028 | 0,0371 |
| Adhesion, migration (17) | | | | | | | |
|  | | | PDPN | Podoplanin (PDPN), | 5,4 | 0,0002 | 0,0112 |
|  |  |  | NRP1 | Neuropilin 1 (NRP1), | 4,4 | 0,0002 | 0,0098 |
|  |  |  | LAMA4 | Laminin, alpha 4 (LAMA4), | 3,3 | 0,0007 | 0,0194 |
|  |  |  | ZEB2 | Zinc finger E-box binding homeobox 2 (ZEB2), | 2,7 | 0,0002 | 0,0094 |
|  |  |  | B4GALT1 | UDP-Gal:betaGlcNAc beta 1,4- galactosyltransferase, polypeptide 1 (B4GALT1), | 2,7 | 0,003 | 0,0026 |
|  |  |  | CEACAM1 | Carcinoembryonic antigen-related cell adhesion molecule 1 (CEACAM1), | 2,6 | 5,4E-06 | 0,0057 |
|  |  |  | ICAM1 | Intercellular adhesion molecule 1 (ICAM1), | 2,5 | 0,0002 | 0,0108 |
|  |  |  | EGFR | Epidermal growth factor receptor (EGFR), | 2,5 | 0,001 | 0,0231 |
|  |  |  | ITGB1 | Integrin, beta 1 (ITGB1), | 2,5 | 0,0009 | 0,0223 |
|  |  |  | ITGA5 | Integrin, alpha 5 (ITGA5), | 2,3 | 0,0005 | 0,0157 |
|  |  |  | LAMA1 | Laminin, alpha 1 (LAMA1), | 2,0 | 0,002 | 0,0335 |
|  |  |  | TPM1 | Tropomyosin 1 (alpha) (TPM1), Transcript variant 5, | -2,1 | 0,025 | 0,1230 |
|  |  |  | CD24 | CD24 molecule (CD24), | -2,99 | 0,0002 | 0,0106 |
|  |  |  | COL5A1 | Collagen, type V, alpha 1 (COL5A1), | -3,2 | 0, 006 | 0,0563 |
|  |  |  | CTGF | Connective tissue growth factor (CTGF), | -3,4 | 0,030 | 0,0305 |
|  |  |  | DLC1 | Deleted in liver cancer 1 (DLC1), | -4,2 | 4,9E-05 | 0,0055 |
|  |  |  | FN1 | Fibronectin 1 (FN1), | -9,25 | 0,0005 | 0,0162 |
| Migration, chemotaxis (6) | | | | | | | |
|  | | | IL6 | Interleukin 6 (IL6) | 75,8 | 0,001 | 0,0263 |
|  |  |  |  | Hypothetical LOC541472 | 46,7 | 0,005 | 0,0527 |
|  |  |  | CXCL16 | Chemokine ligand 16 (CXCL16) | 2,74 | 9,1E-06 | 0,0041 |
|  |  |  | IL6R | Interleukin 6 receptor (IL6R) | 2,68 | 0,002 | 0,0286 |
|  |  |  | IL1B | Interleukin 1, beta (IL1B) | 2,6 | 0,004 | 0,0670 |
|  |  |  | VEGFA | Vascular endothelial growth factor A (VEGFA) | 2,38 | 0,0002 | 0,0157 |
| Chemotaxis, adhesion (1) | | | | | | | |
|  | | | KAL1 | Kallmann syndrome 1 sequence (KAL1) | -3,74 | 0,01 | 0,0743 |

**F.C.**: fold change
